# Prevalence and association of HIV and tuberculosis status in older adults in South Africa: an urgent need to escalate the scientific and political attention to aging and health

**DOI:** 10.3389/fpubh.2024.1245553

**Published:** 2024-03-15

**Authors:** Monica Ewomazino Akokuwebe, Godswill Nwabuisi Osuafor, Erhabor Sunday Idemudia

**Affiliations:** ^1^Faculty of Humanities, North-West University, Mafikeng, South Africa; ^2^Department of Population Studies and Demography, North-West University, Mafikeng, South Africa

**Keywords:** factors, HIV, older adults, sociodemographic, TB

## Abstract

**Objectives:**

This study examined the prevalence and sociodemographic factors among older adults with HIV and TB status in South Africa.

**Methods:**

This data was cross-sectional and obtained from the 2019 General Household Surveys in South Africa. Adults 50 years and over with reported HIV and TB status were included (*N* = 9,180,047). We reported statistical analyses of the descriptive, Chi-square and Fisher’s exact tests, and binary logistic regression.

**Results:**

The study has found a prevalence rate of HIV to be 5.3% and TB to be 2.9% among older adults aged 50 years and above in South Africa. However, the study found HIV and TB to be highest among older adults residing in Gauteng, KwaZulu-Natal and Eastern Cape provinces. For HIV status, the female gender [AOR = 0.80*, CI 95% = 0.80–0.80] and secondary education [AOR = 0.57, CI 95% = 0.56–0.58] have lower odds of association among older adults with HIV. Regarding TB status, primary education [AOR = 1.08*, CI 95% = 1.06–1.10] and diabetes [AOR = 1.87*, CI 95% = 1.82–1.91] have lower likelihoods of associations among older adults with TB.

**Conclusion:**

There is an urgent need to escalate scientific and political attention to address the HIV/TB burden in older adults and, public health policymakers need to take cognizance of the interdependence of inequality, mobility, and behavioural modification among this high-risk population.

## Introduction

Regarding the phenomenon of aging populations, several studies that have emerged on aging populations and health have mainly focused on the changing demographic factors contributing to the patterns of aging and the economic and social ramifications of health systems in older population (1–3). According to studies conducted in high-income nations like North America and Europe, older persons have distinct health demands than younger and middle-aged people (4). However, much less is known about these needs and care gaps in developing countries (5). According to the 2011 South African census, nearly 40 % of older persons live in poverty and have severe unemployment. Additionally, older adults now have significant care and financial responsibilities as they use their retirement pension as well as leisure time to support their children and grandchildren, with a noticeable effect on their general well-being as a direct consequence of the adverse outcomes of the Human Immunodeficiency Virus (HIV) and Tuberculosis (TB) (6–8). Disease diagnosis, treatment, and prevention have imposed an enormous burden on older adult people who are affected, as well as the healthcare system. Understanding this phenomenon’s health impact on the aging population is vital for planning in the health systems, yet there is insufficient morbidity data on HIV/TB among older adults in South Africa.

Concerning these data gaps, various research collaborations have concentrated on collecting cross-sectional, longitudinal and cross-country data on aging in low-and middle-income countries (LMICs), such as in South Africa. South Africa now has a rising proportion of older adults infected with HIV and TB and is home to the most extensive antiretroviral program in the world (9, 10). Globally, there is an increasing number of individuals infected with HIV, including those in the age cohorts of 50 years or older, with increasing numbers being diagnosed and living with HIV and TB co-infections (2, 11). In South Africa, more than 6.3 million persons lived with HIV and TB in 2021, of whom around 3.1 million were receiving antiretroviral therapy (ART) (2, 12). Several studies have reported a dual burden of HIV and TB, with an estimation of 60% of persons diagnosed with TB being co-infected with HIV (13). For instance, assessments from 2012 showed that the average prevalence of HIV in those over 50 years old is 7.6% (95% CI 6.5–8.8), while the prevalence of HIV infection in age cohorts of 15–49 years is anticipated to decrease by half, and in the next 30 years, HIV infection in older adults aged over 50 years is predicted to double in the next 30 years (14, 15).

Furthermore, in South Africa, older persons are not specifically targeted for HIV and TB prevention and testing, and medical providers, especially community health workers, may fail to suspect or test for infections in this “hard-to-reach population,” resulting in ignored, delayed or postponed diagnosis (16–20). Also, a lack of non-use of condoms has been reported among older adults, and studies have shown that marital status plays a significant role in the non-use of condoms among older married adults (3, 7). Again, due to religious beliefs, societal values or cultural roots, condom use is uncommon among older adults who are married, or cohabiting, as sexual activities and condom use among older persons will be frowned upon, which may lead to sexual stigmatization (8, 11). An urgent action is required to implement evidence-based priorities to manage the HIV and tuberculosis epidemics, as this would help to reduce HIV and TB cases among older persons in South Africa. It is crucial to promote comprehensive access to health services that ensure the health needs of older adults with HIV and TB infections so that SDG 3.3 can be achieved (which mentions TB alongside HIV explicitly and acquired immunodeficiency syndrome [AIDS] and malaria) (21–24). Therefore, it is necessary to provide an empirical conclusion to promote a comprehensive knowledge and interpretation of healthcare barriers as perceived in older adults. This study, therefore, sought to examine the prevalence of HIV and TB epidemics in the provinces, and to determine the baseline sociodemographic factors associated with older adults with HIV and TB in South Africa. A further objective was to find associations with the predicting factors within the older adult cohorts.

## Materials and methods

### Study setting and population characteristics

The study setting is the Republic of South Africa (RSA), in the southern African region, renowned for its varied topography, natural beauty and cultural diversity (25). The country with 471,445 sq. km has a heterogeneous cultures, each of which are predominant in the nine provinces, and with an upper-middle-income economy (26). The South Africa’s economy grew in the early era of the 21st century, but declined recently, as the country experienced high unemployment, and widespread poverty and inequality. With a population of 60.6 million in the year (27), about 51.1% (30.5 million) of the population is female, about 28.6% is younger than 15, while approximately 9.1% (54 million) is aged 60 years and older (27). Furthermore, comparable projections have shown that total HIV prevalence is estimated at 13.0% among the South African population, with approximately 7.8 million people living with HIV (PLWHIV), and an estimated 18.7% of the population is HIV positive (28, 29). Also, South Africa is one of the 30 high burden TB countries contributing 87% of the estimated incident TB cases around the world, as 3% of cases globally are attributed to South Africa alone (12). Thus, among these 30 high burden TB nations, South Africa is among the 14 countries with the highest burden of TB, TB/HIV and multi-drug resistant TB (MDR-TB) (10–12). The HIV co-infection rate in reported TB patients in South Africa was 59% in the 2019 Global TB report, highlighting the ongoing virus relevance to the TB epidemic (12). Therefore, HIV infection is a critical factor in the TB epidemic. Persons living with HIV have higher chances of being susceptible to TB, with a higher mortality rate. This summation underlines the vital aspects of the political will, political economy, culture, and contextual influences on their lifestyles and behaviors.

### Data source and sampling procedures

This study used national and cross-sectional data on HIV and TB epidemics among older adult populations obtained from the 2019 General Household Surveys (GHS) in South Africa. The 2019 General Household Surveys (GHS) was conducted in South Africa between December 12, 2019, and January 1, 2020, in nine provinces (and metropolitan municipalities, where this applies). The choice of using the 2019 GHS for this study was owing to the relevant factors that are present in the datasets, and the GHS is conducted countrywide in both urban and rural provinces annually (30). Also, the survey targets and measures the living conditions of households in each of the provinces in South Africa, hence its national representativeness. From the 2015 General Household Survey (GHS) uses a Master Sample (MS) frame developed in 2013 as a general-purpose sampling frame to be used for all Stats SA household-based surveys. This MS has design requirements that are practically consistent with the GHS. The 2013 Master Sample is based on information collected during the 2011 Census conducted by Stats SA. In preparation for Census 2011, the country was divided into 103,576 enumeration areas (EAs).

The census EAs, together with the auxiliary information for the EAs, were used as the frame units or building blocks for the formation of primary sampling units (PSUs) for the Master Sample, since they covered the entire country, and had other information that is Master Sample, since they covered the entire country, and had other information that is crucial for stratification and creation of PSUs. There are 3,324 primary sampling units (PSUs) in the Master Sample, with an expected sample of approximately 33,000 dwelling units (Dus). The number of PSUs in the current Master Sample (3,324) reflect an 8.0% increase in the size of the Master Sample compared to the previous Master Sample (which had 3,080 PSUs in year 2008). The larger Master Sample of PSUs was selected to improve the precision (smaller coefficients of variation, known as CVs) of the GHS estimates. The Master Sample is designed to be representative at provincial level and within provinces at metro/non-metro levels. Within the metros, the sample is further distributed by geographical type. The three geography types are urban, tribal and farms. This implies, for instance, that within a metropolitan area, the sample is representative of the different geography types that may exist within that metro. The sample for the GHS is based on a stratified two-stage design with probability proportional to size (PPS) sampling of PSUs in the first stage, and sampling of dwelling units (Dus) with systematic sampling in the second stage.

After allocating the sample to the provinces, the sample was further stratified by geography (primary stratification), and by population attributes using Census 2011 data (secondary stratification). In selecting the sample for the survey, a multi-stage sampling approach was employed based on a stratified two-stage design. The primary stage of this selection method involved the sampling and selection of clusters (i.e., enumeration areas [EAs]) using the methodological approaches of the probability proportional to size (PPS) and the proportional sampling units (PSUs) (30). The sampling of dwelling units (DUs) within the selected EAs with systematic sampling was carried out in the subsequent phase. Subsequently assigning the sample to the provinces, the sample was further stratified by provincial individualities (primary stratification) and by population characteristics using the Census 2011 data (secondary stratification) (31). The population estimates for 2019 GHS was 58,428,891 and the household estimates was 17,162,983. In this study, the sample size comprised of older adult male and female respondents aged 50–75^+^ years with comprehensive data on all the variables of interest (*N* = 9,180,047) (32). The national response rate for the survey was 39.4%, and the highest response rate (56.5%) was recorded in Limpopo and the lowest in Gauteng (29.7%) provinces (30).

### Data weighting

Sample weights were created for the 2019 GHS in order to account for the design weights and non-responses from the survey data. Thus, sampling weights for the sampled households were created to account for respondents’ responses that may adequately be expanded to represent the entire civilian population of South Africa (30). The Demographic Analysis Division’s mid-year population projections was utilized as the standard benchmark, and age groupings was stratified into 5-year age range (0–4, 5–9, 10–14, 55–59, 60–64, and 65) and age at the provincial level (0 to 14, 15 to 34, 35 to 64, and 65 and beyond) (30). All household members were intended to have the same final weight when the calibrated weights were put together (30, 31). Cells identified by cross-classification of age, gender, and race were subjected to national and provincial population controls. The records that could not be weighted owing to item non-response according to age, demographic group, or sex were excluded from the dataset (30, 31). In order to analyze patterns and create models to estimate the number of households each year, the databases of the 1996, 2001, 2007, and 2011 censuses were employed (30). The weighting structure used tables showing the predicted household head distribution across various age brackets, broken down by population and province.

### Definition of variables

#### Outcome variable

The outcome variable is reported HIV and TB status, gotten from respondents’ responses who are adults aged 50 years and over. The outcome variable has a categorical outcome with two levels using the binary code “1 = yes” and “0 = no” were created. The outcome variable was derived from respondents’ responses such as, “whether they have been found with HIV” during the survey? or “whether they have been found with TB” during the survey?, whether respondents were told that he or she had HIV by doctor/nurse/other healthcare workers at a clinic/hospital/private practice?” or whether respondents were told that he or she had TB by doctor/nurse/other healthcare workers at a clinic/hospital/private practice?” and if their responses were either “no” or “yes.” Thus, if the respondents with HIV answered yes to the questions mentioned above, they were coded as “1”, otherwise coded as “0”. Hence, if the respondents with TB answered yes to the aforementioned questions, were coded as “1”, otherwise coded as “0”. Although the question involves respondents who has/had, given that TB is a chronic infectious disease, therefore it infers that those who had mostly likely still have it because it takes a long time for people to recover from these type of disease burden (33, 34). The respondents who reported that they have HIV or TB were asked to provide proof of their clinical diagnosis (hospital documents and medications).

#### Independent variables

In this study, factors at the individual-and contextual-level were considered. These factors were considered because they had a statistically significant association with older persons who had HIV and TB in earlier studies (34–36). The individual-level factors included age (50–54, 55–59, 60–64, 65–69, 70–74, 75+), population group (African/Black, Colored, Indian/Asian, White), marital status (married, never married, divorced, widowed), gender (male, female), educational level (no education, primary, secondary/higher), and business activities (yes, no). The contextual variables were sex (male, female), geographical location (urban, rural), health status (excellent, good, fair, poor), diabetes (yes, no), cancer (yes, no), and medical aid scheme (yes, no).

### Statistical analyses

The data were analyzed with STATA version 14.2 for MacOS. Three basic steps were followed to analyze the data. The first step utilize descriptive statistics to describe the sample characteristics of the respondents (frequency and percentages) (Table 1). The second step of analysis was a bivariate analysis that ensures the cross-tabulation of all the independent variables against the outcome variable using the Chi-square and Fisher’s exact tests and potential variables for the regression analysis were identified (Table 2). The third step of analysis was binary logistic regression that include variables that were statistically significant at the bivariate level of analysis (Table 3). In the binary logistic regression analysis, clusters were considered a random effect to account for the unexplained variability at the community level and four models were fitted for older adults with HIV and TB status separately (Table 3). Model I is the random intercept of the empty model with no predictors. Model II include the individual-level variables and Model III only incorporate the contextual-level variables. While Model IV involves both the individual-level and contextual-level factors. Adjusted odds ratios (AOR) and their associated 95% confidence intervals (CIs) were presented for all Models, fitted into the Models by a STATA command ‘melogit’ that identifies predictors with the outcome variable (Table 3). For model assessment, the log-likelihood ratio (LLR) was employed and sample weight was applied to address related concerns of over-and under-sampling. However, the SVY command was used to account for the complex survey design and generalizability of the findings. The statistical significance for all tests was set at *ρ* < 0.05.

**Table 1:** Socio-demographic characteristics of older adults aged 50–75^+^ years with and without HIV and TB in South Africa (*n* = 9,180,047).

Socio-demographic characteristic	*N*	Frequency (*N* = 9,180,047)	Percentage (100.00)
		*n*	%
Age	50–54	2,380,545	25.93
	55–59	2,042,083	22.24
	60–64	1,623,918	17.69
	65–69	1,244,219	13.55
	70–74	855,630	9.32
	75^+^	1,033,652	11.26
Marital status	Married	4,536,828	49.42
	Never Married	1,892,737	20.62
	Divorced	566,708	6.17
	Widowed	2,183,774	23.79
Population group	African/Black	6,121,751	66.69
	Colored	1,000,353	10.90
	Indian/Asian	350,949	3.82
	White	1,706,994	18.59
Gender	Male	3,906,737	42.56
	Female	5,273,309	57.44
Educational attainment	No education	1,048,881	11.43
	Primary	2,492,551	27.15
	Secondary/Higher	5,638,615	61.42
Geographical location	Urban	6,158,956	67.09
	Rural	3,021,090	32.91
Any business activities	Yes	807,093	8.79
	No	8,372,954	91.21

**Table 2:** Bivariate analysis of the population characteristics by outcome variables among older persons who reported HIV and TB status in South Africa (*n* = 9,180,047).

Population characteristics	HIV status					TB status				
	No, *n* = 8,843,271		Yes, *n* = 336,776		Total, *N* = 9,180,047	No, *n* = 9,096,355		Yes, *n* = 83,692		Total, *N* = 9,180,047
	*N*	%	*N*	%		*N*	%	*N*	%	
Age group			χ^2^ = 179.0011; *p* = 0.000			χ^2^ = 7.2508; *p* = 0.203				
50–54	2,242,710	94.21	137,835	5.79	2,380,545	2,357,789	99.04	22,756	0.96	2,380,545
55–59	1,926,292	94.33	115,791	5.67	2,042,083	2,018,771	98.86	23,313	1.14	2,042,083
60–64	1,578,281	97.19	45,637	2.81	1,623,918	1,607,643	99.00	16,274	1.00	1,623,918
65–69	1,217,793	97.88	26,426	2.12	1,244,219	1,232,253	99.04	11,966	0.96	1,244,219
70–74	849,632	99.30	5,999	0.70	855,630	849,518	99.29	6,113	0.71	855,630
75+	1,028,564	99.51	5,088	0.49	1,033,652	1,030,382	99.68	3,270	0.32	1,033,652
Marital status			χ^2^ = 219.7989; *p* = 0.000			χ^2^ = 9.5142; *p* = 0.023				
Married	4,450,816	98.10	86,012	1.90	4,536,828	4,501,141	99.21	35,687	0.79	4,536,828
Never Married	1,735,620	91.70	157,117	8.30	1,892,737	1,864,454	98.51	28,284	1.49	1,892,737
Divorced	539,475	95.19	27,232	4.81	566,706	561,074	99.01	5,633	0.99	566,706
Widowed	2,117,360	96.96	66,414	3.04	2,183,774	2,169,687	99.35	14,088	0.65	2,183,774
Population group			χ^2^ = 169.8998; *p* = 0.000			χ^2^ = 20.3397; *p* = 0.000				
African/Black	5,788,937	94.56	332,814	5.44	6,121,751	6,047,393	98.79	74,358	1.21	6,121,751
Colored	997,159	99.68	3,195	0.32	1,000,353	992,974	99.26	7,380	0.74	1,000,353
Indian/Asian	350,949	100.00	-	0.00	350,949	350,949	100.00	0	0.00	350,949
White	1,706,227	99.96	767	0.04	1,706,994	1,705,040	99.89	1,954	0.11	1,706,994
Gender			χ^2^ = 0.0032; *p* = 0.955			χ^2^ = 50.1063; *p* = 0.000				
Male	3,755,477	96.13	151,260	3.87	3,906,737	3,844,939	98.42	61,799	1.58	3,906,737
Female	5,087,794	96.48	185,516	3.52	5,273,309	5,251,416	99.58	21,893	0.42	5,273,309
Educational attainment			χ^2^ = 47.7713; *p* = 0.000			χ^2^ = 16.0941; *p* = 0.000				
No education	996,018	94.96	52,863	5.04	1,048,881	1,035,043	98.68	13,839	1.32	1,048,881
Primary	2,352,020	94.36	140,531	5.64	2,492,551	2,455,730	98.52	36,820	1.48	2,492,551
Secondary/Higher	5,495,233	97.46	143,381	2.54	5,638,615	5,605,582	99.41	33,033	0.59	5,638,615
Geographical location			χ^2^ = 18.1792; *p* = 0.000			χ^2^ = 2.4136; *p* = 0.120				
Urban	5,974,117	97.00	184,839	3.00	6,158,956	6,113,395	99.26	45,561	0.74	6,158,956
Rural	2,869,154	94.97	151,936	5.03	3,021,090	2,982,960	98.74	38,130	1.26	3,021,090
Business activities			χ^2^ = 2.7385; *p* = 0.098			χ^2^ = 2,7,110; *p* = 0.100				
Yes	788,103	97.65	18,989	2.35	807,093	802,502	99.43	4,591	0.57	807,093
No	8,055,168	96.20	317,786	3.80	8,372,954	8,293,853	99.06	79,101	0.94	8,372,954
Health status			χ^2^ = 61.9589; *p* = 0.000			χ^2^ = 55.1765; *p* = 0.000				
Excellent	990,900	98.64	13,658	1.36	1,004,559	1,001,636	99.71	2,922	0.29	1,004,559
Good	5,403,426	96.89	173,608	3.11	5,577,033	5,540,589	99.35	36,444	0.65	5,577,033
Fair	1,880,616	94.48	110,382	5.54	1,990,997	1,966,267	98.76	24,730	1.24	1,990,997
Poor	568,329	93.56	39,128	6.44	607,457	5,878,623	96.77	19,595	3.23	607,457
Diabetes			χ^2^ = 19.3757; *p* = 0.000			χ^2^ = 3.0581; *p* = 0.080				
Yes	1,255,060	98.21	22,810	1.79	1,277,871	1,270,668	99.44	7,203	0.56	1,277,871
No	7,588,211	96.03	313,965	3.97	7,902,176	7,825,687	99.03	76,489	0.97	7,902,176
Cancer			χ^2^ = 0.1305; *p* = 0.718			χ^2^ = 4.7637; *p* = 0.029				
Yes	118,373	97.06	3,585	2.94	121,958	118,385	97.07	3,573	2.93	121,958
No	8,724,898	96.32	333,191	3.68	9,058,089	8,977,969	99.12	80,119	0.88	9,058,089
Medical aid scheme			χ^2^ = 88.8317; *p* = 0.000			χ^2^ = 18.6860; *p* = 0.000				
Yes	2,334,018	99.47	12,411	0.53	2,346,429	2,341,527	99.79	4,902	0.21	2,346,429
No	6,509,253	95.25	324,365	4.75	6,833,618	6,754,828	98.85	78,790	1.15	6,833,618

*χ*^2^ = Chi-square notation; *ρ* = probability of level of significance.

**Table 3:** Multi-level logistic regression of individual and contextual factors associated with HIV and TB infections among older persons in South Africa.

Variables	HIV Status			TB Status		
	Model I	Model II	Model III	Model I	Model II	Model III
Individual variables						
Age group						
50–54	RC		RC	RC		RC
55–59	0.93 (0.92–0.93)		0.91 (0.90–0.92)	1.12* (1.10–1.14)		1.13* (1.11–1.15)
60–64	1.41* (0.40–2.41)		0.37* (0.36–0.37)	0.93 (0.91–0.95)		0.83 (0.82–0.85)
65–69	0.29 (0.29–0.29)		0.25 (0.25–0.26)	0.87 (0.85–0.89)		0.71 (0.69–0.73)
70–74	0.09 (0.09–0.09)		0.08 (0.07–0.08)	0.66 (0.64–0.68)		0.44 (0.43–0.45)
75+	0.06 (0.06–0.06)		0.05 (0.05–0.05)	0.30 (0.30–0.31)		0.20 (0.20–0.21)
Marital status						
Married	RC		RC	RC		RC
Never Married	3.57* (3.54–3.61)		2.72* (2.70–2.75)	1.78 (1.75–1.81)		1.32 (1.30–1.34)
Divorced	2.61 (2.57–2.64)		2.27 (2.24–2.31)	1.53 (1.49–1.58)		1.29 (1.25–1.33)
Widowed	2.33 (2.31–2.36)		1.85 (1.83–1.87)	1.36* (1.34–1.39)		1.10* (1.07–1.12)
Gender						
Male	RC		RC	RC		RC
Female	0.80 (0.80–0.81)		0.80* (0.80–0.80)	0.23* (0.23–0.24)		0.23 (0.23–0.24)
Education						
No education	RC		RC	RC		RC
Primary	0.82 (0.81–0.83)		0.92 (0.91–0.93)	0.88 (0.87–0.90)		1.08* (1.06–1.10)
Secondary/Higher	0.35 (0.35–0.35)		0.57 (0.56–0.58)	0.35 (0.35–0.36)		0.65* (0.64–0.67)
Business activities						
Yes	RC		RC	RC		RC
No	1.77* (1.74–1.80)		1.52* (1.50–1.55)	1.84* (1.79–1.90)		1.47* (1.42–1.51)
Contextual variables						
Geographical location						
Urban		RC	RC		RC	RC
Rural		1.23* (1.22–1.24)	1.27* (1.27–1.29)		1.28* (1.27–1.30)	1.38* (1.36–1.40)
Health status						
Excellent		RC	RC		RC	RC
Good		2.10* (2.07–2.14)	2.28 (2.24–2.32)		2.04 (1.96–2.12)	2.27 (2.19–2.36)
Fair		3.71 (3.64–3.78)	4.95 (4.86–5.05)		3.70 (3.56–3.84)	4.77* (4.59–4.96)
Poor		4.33* (4.25–4.42)	6.10* (5.98–6.23)		9.48* (9.11–9.86)	12.03* (11.55–12.52)
Diabetes						
Yes		RC	RC		RC	
No		2.81* (2.77–2.85)	2.14* (2.11–2.17)		2.41* (2.35–2.47)	1.87* (1.82–1.91)
Cancer						
Yes		RC	RC		RC	RC
No		1.11 (1.08–1.15)	0.66* (0.63–0.68)		0.32* (0.31–0.33)	0.24 (0.23–0.25)
Medical scheme						
Yes		RC	RC		RC	RC
No		7.48* (7.34–7.62)	4.51* (4.42–4.59)		4.26* (4.14–4.39)	3.11* (3.02–3.21)
Contextual level						
Variance (SE)	0.0004113	0.0000188	0.0000708	0.0285024	0.0000363	0.0001401
OR (95% CI)	0.04 (0.04–0.05)	0.001 (0.001–0.009)	0.003 (0.003–0.004)	0.02 (0.02–0.03)	0.001 (0.002–0.003)	0.004 (0.004–0.005)
*N*	9,180,047	9,180,047	9,180,047	9,180,047	9,180,047	9,180,047

Model I: individual-level variables; Model II: contextual variables; Model III: final model adjusted for individual and contextual variable; RC, Reference Category; ^*^significance test – *α* < 0.05.

### Ethical approval

All data were obtained from the 2019 GHS datasets and signed informed consent forms were retrieved from the respondents before their participation in the interviews (35). An approval for data usage from the GHS repository of the household level data of the 2019 General Household Survey was obtained. Relevant ethical guidelines following the Declaration of Helsinki were adopted. The GHS follows the ethical principles of the protection of respondents’ privacy and the survey was ensured to comply with the ethical principles relating to human subjects. This study uses a secondary data, and as such no further approval was required. Further information about the usage and ethical standards of the 2019 GHS data are available at https://www.datafirst.uct.ac.za/dataportal/index.php/catalog/852/download/11753.

## Results

### Socio-demographic characteristics of older adults with HIV and TB in South Africa

Table 1 shows the weighted sample of 9,180,047 and the socio-demographic characteristics of older adults with HIV and TB in South Africa. About 25.9% of the older adults with HIV and TB were aged 50–54. More than half (57.4%) were females, 49.4% were married, 66.7% were African/Black, and 61.4% of older adults with HIV and TB have attained secondary/higher education. A greater percentage of the older adults with HIV and TB were not engaged in business activities, and more than half (67.1%) were residing in urban locations (Table 1).

### Prevalence of HIV by age among older adults aged 50–75^+^ in South Africa

**Figure 1:**
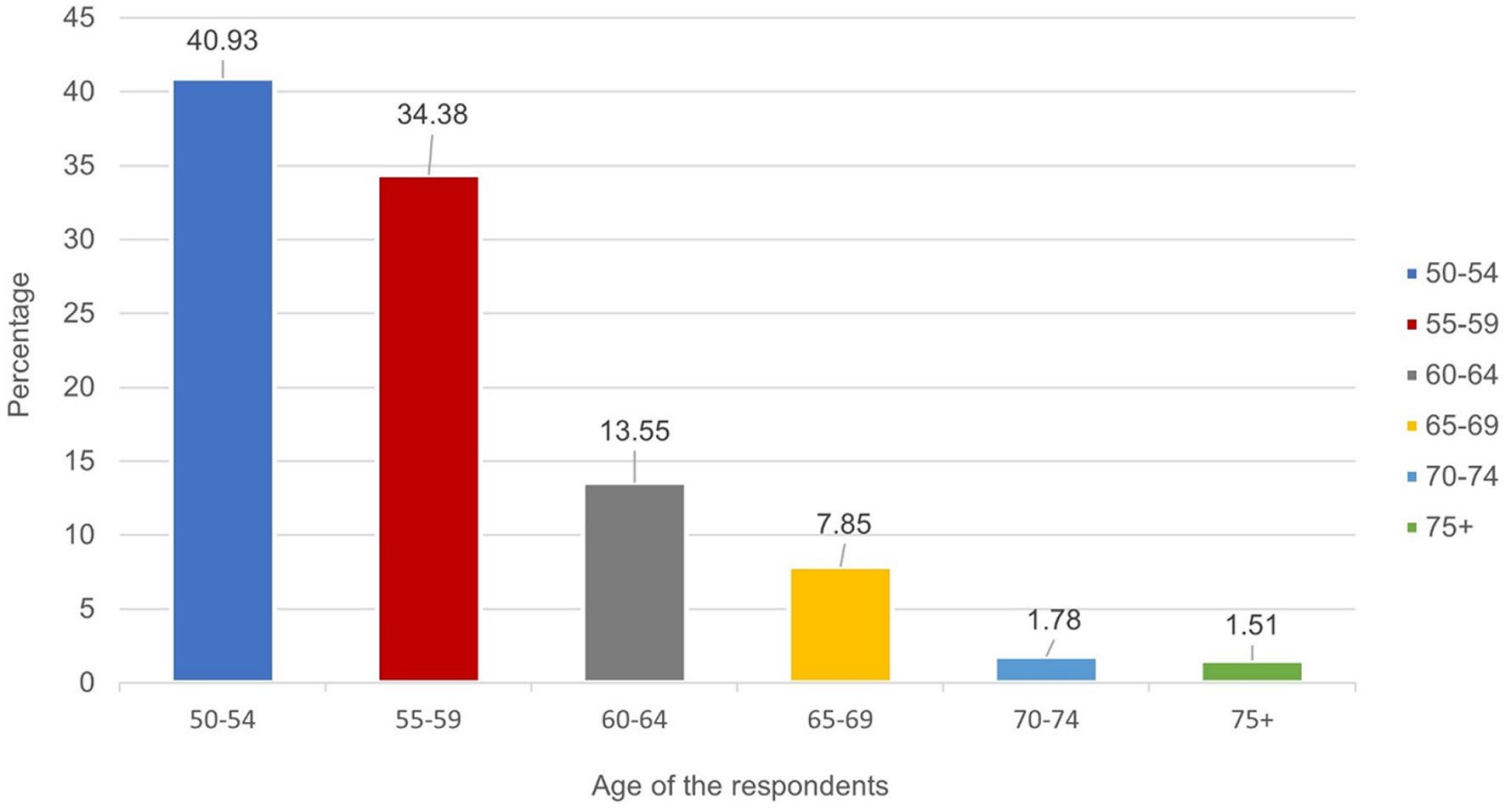
HIV prevalence by age group (50–75^+^) in South Africa.

The prevalence of HIV by age among adults aged 50 years and older was shown in Figure 1. HIV was found to be prevalent among older adults aged 50–54 years (40.93%), 55–59 years (34.38%), 60–64 years (13.55%) and 65–69 years (7.85%). The study also found lower prevalence of HIV among older adults aged 70–74 years (1.78%) and 75^+^ years (1.51%) (Figure 1).

### Prevalence of TB by age among older adults aged 50–75^+^ in South Africa

**Figure 2:**
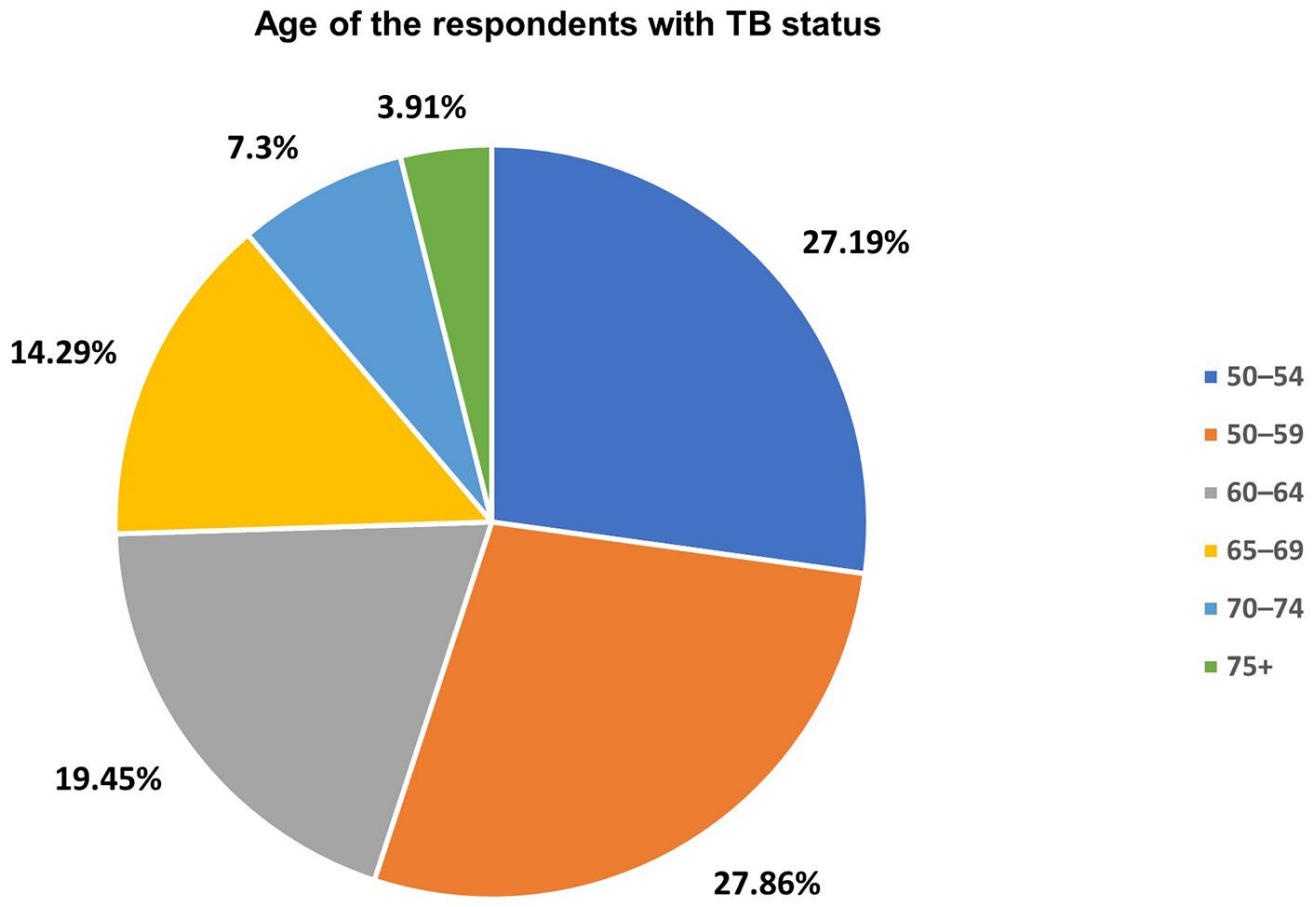
TB prevalence by age group (50–75^+^) in South Africa.

The prevalence of TB by age among adults aged 50 years and older was shown in Figure 2. TB was found to be prevalent among older adults aged 50–54 years (27.19%), 55–59 years (27.86%), 60–64 years (19.45%) and 65–69 years (14.29%). The study also found lower prevalence of TB among older adults aged 70–74 years (7.30%) and 75^+^ years (3.91%) (Figure 2).

### Prevalence of HIV by province among older adults aged 50–75^+^ in South Africa

**Figure 3:**
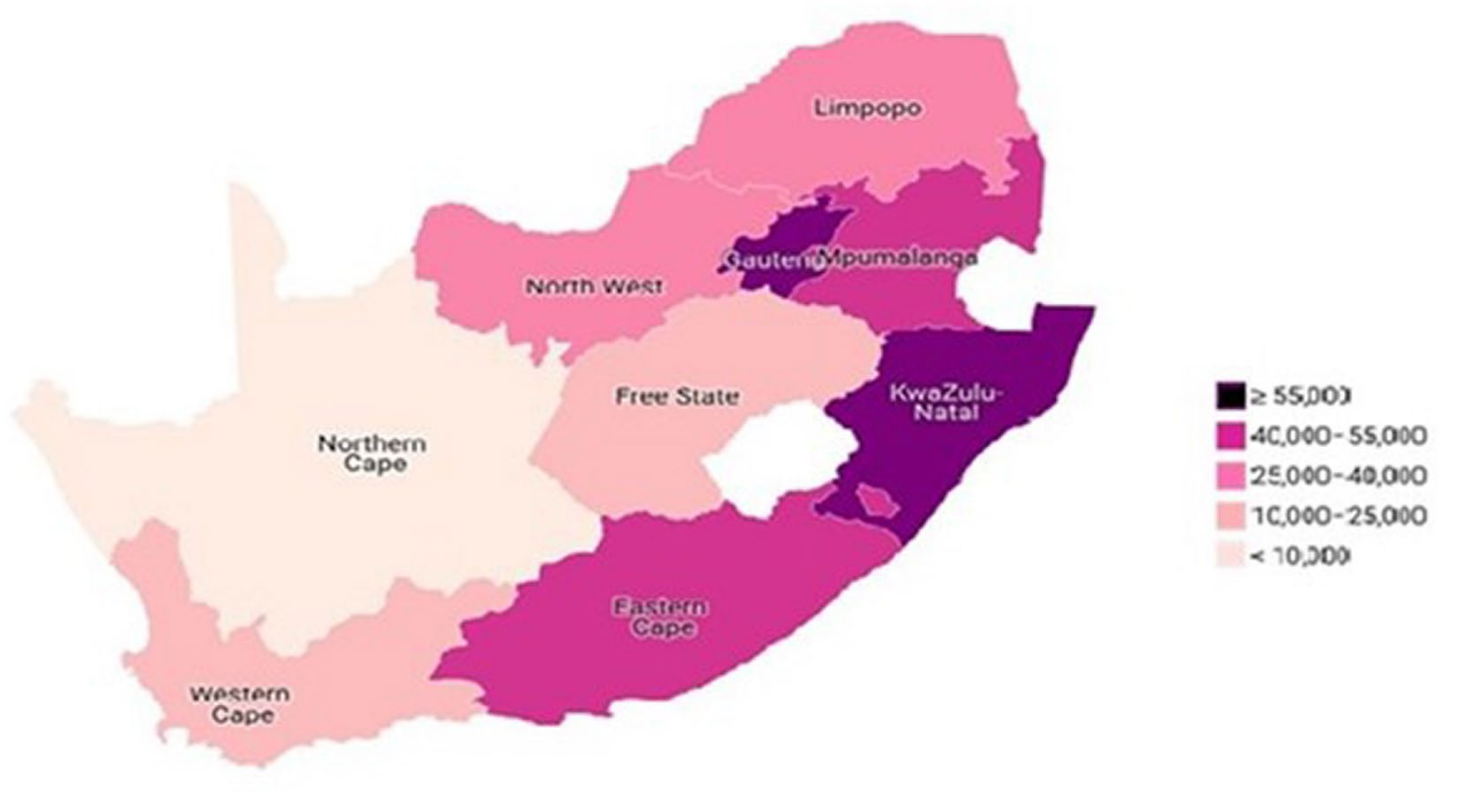
HIV prevalence by province among older adults (50–75^+^) in South Africa.

**Figure 4:**
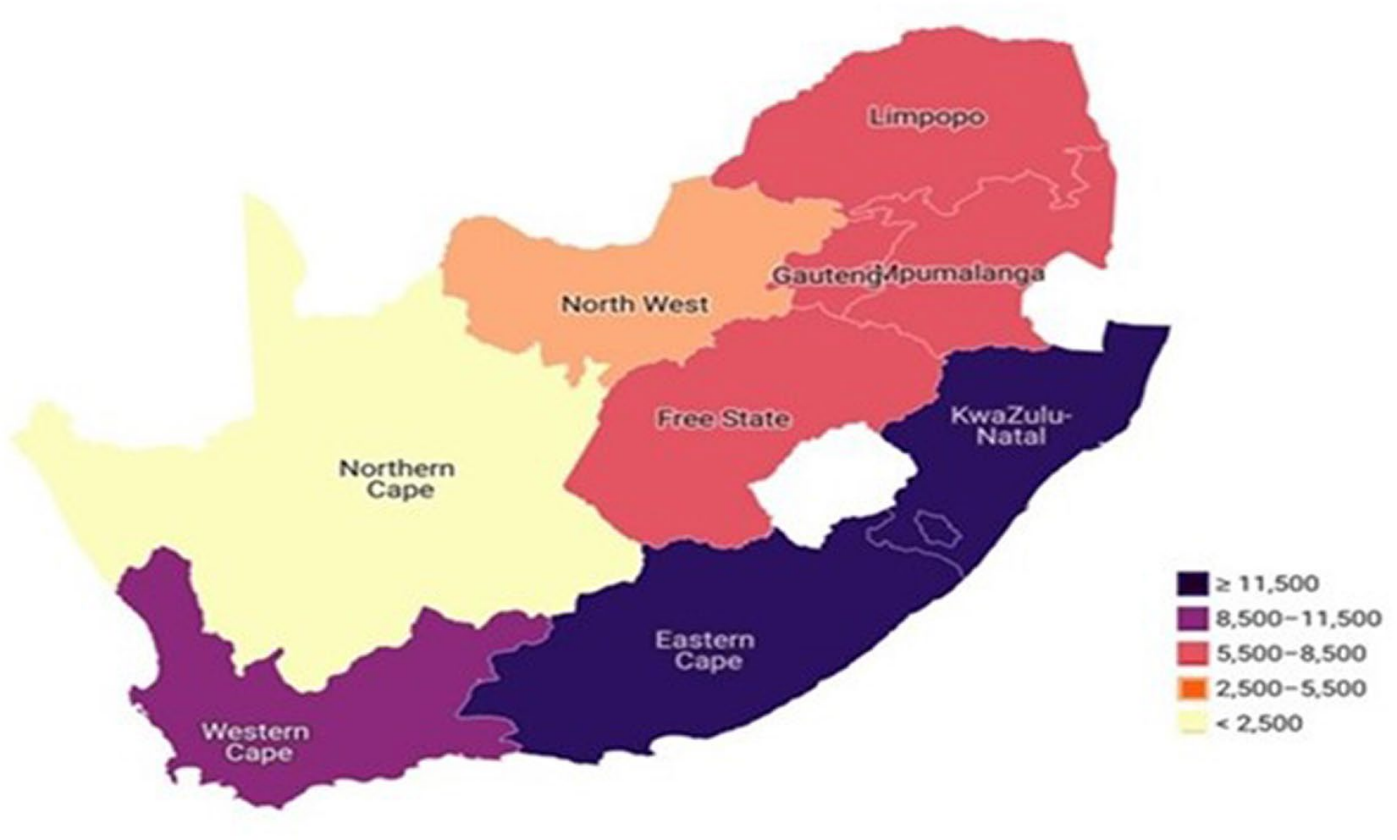
TB status prevalence by province among older adults (50–75^+^) in South Africa.

The prevalence of HIV among adults aged 50 years and older was 5.3%. Figure 3 showed the prevalence of HIV among older adults aged 50–75^+^ in the different provinces of South Africa. The findings revealed that more than 55,000 older adults with HIV are residing in Gauteng and KwaZulu-Natal provinces, while between 40,000 and 55,000 older adults with HIV reside in Eastern Cape and Mpumalanga provinces (Figure 3). Also, about 25,000 and 40,000 older adults with HIV were residing in North-West and Limpopo. Similarly, between 10,000 and 25,000 older adults with HIV are residing in Free State and Western Cape provinces. Further, fewer than 10,000 older adults with HIV are residing in Northern Cape (Figure 4).

### Prevalence of TB status by province among older adults aged 50–75^+^ in South Africa

The prevalence of TB among adults aged 50 years and older was 2.9%. Figure 4 reports the prevalence of TB status by province among older adults aged 50–75^+^ in South Africa. In this study findings, the Choropleth map showed that more than 11,500 older adults with TB were found residing in Eastern Cape and KwaZulu-Natal provinces, while between 8,500 and 11,500 older adults with TB were residing in Western Cape Province (Figure 4). Remarkably, between 5,500–8,500 of older adults with TB were found residing across four major provinces: Gauteng, Mpumalanga, Free State and Limpopo. However, between 2,500–5,500 older adults with TB were found residing in the North West province and fewer than 2,500 older adults with TB were residing in Northern Cape Province (Figure 4).

### Factors associated with older adults with HIV and TB in South Africa

Table 2 shows the findings of the bivariate analysis of socio-demographic factors associated with older adults with HIV and TB in South Africa. The chi-square analysis of the HIV status showed explanatory variables such as age (50–54 years old: 5.79%, *ρ* < 0.000), marital status (never married: 8.30%, *ρ* < 0.000), population group (African/Black: 5.44%, *ρ* < 0.000), educational status (no education: 5.04%, *ρ* < 0.000; primary: 5.64%, *ρ* < 0.000), geographical location (rural: 5.03%, *ρ* < 0.000), health status (fair: 5.54%, *ρ* < 0.000; poor: 6.44, *ρ* < 0.000), diabetes (no: 3.97%, *ρ* < 0.000) and medical aid scheme (no: 4.75%, *ρ* < 0.000) have demonstrated statistically significant associations with HIV status among older adults in South Africa (Table 2). As for older adults with TB, the bivariate analysis of the socio-demographic factors, the chi-square findings showed that explanatory factors such as marital status (never married: 1.49%, *ρ* = 0.023), population group (Black African: 1.21%, *ρ* < 0.00 0), gender (male: 1.58%, *ρ* < 0.000), education attainment (no: 1.32, *ρ* < 0.000; primary: 1.48%, *ρ* < 0.000), health status (fair: 1.24%, *ρ* < 0.000; poor: 3.23%, *ρ* < 0.000), cancer (yes: 2.93%, *ρ* < 0.029), and medical aid scheme (no: 1.15%, *ρ* < 0.000) demonstrated statistical significant associations with TB status among older adults in South Africa (Table 2).

### Multilevel logistic regression of the individual and contextual factors associated with HIV and TB infections among older persons in South Africa

Table 3 shows the multilevel logistic regression results of the Model IV of the final model adjusted for individual-level and contextual factors associated with HIV and TB infections among older adults in South Africa. Reporting individual-level factors, older adults aged 60–64 years [AOR = 1.41, 95% CI = 0.40–2.41, *ρ* < 0.05] had higher odds of being associated with HIV than those aged 50–54. The study showed that never-married older adults [AOR = 3.57, 95% CI = 3.54–3.61, *ρ* < 0.05] were more likely to have HIV compared to older adults who had married (Table 3). With gender, compared to males, females [AOR = 0.80, 95% CI = 0.80–0.80, *ρ* < 0.05] had lower odds of being associated with HIV. Compared with older adults with no education, those with primary [AOR = 0.92, 95% CI = 0.91–0.93, *ρ* < 0.05] and secondary [AOR = 0.57, 95% CI = 0.56–0.58, *ρ* > 0.05] education had the lowest odds of being associated with HIV. Regarding the contextual factors associated with HIV status among older adults in South Africa, those who resided in a rural geographical location [AOR = 1.27, 95% CI = 1.27–1.29, *ρ* < 0.05] were more likely to be associated with having HIV, compared with urban older adults (Table 3).

Likewise, with regards to TB status among older adults with individual-level factors, the study showed that older adults aged 55–59 years [AOR = 1.13, 95% CI = 1.11–1.22, *ρ* < 0.05] were more likely to be associated with having TB compared to their counterparts who are aged 50–54 years old. Compared to those who are married, older adults who are widowed [AOR = 1.36, 95% CI = 1.34–1.39, *ρ* < 0.05] had higher odds of being associated with having TB. Older adults who are female [AOR = 0.23, 95% CI = 0.23–0.24, *ρ* > 0.05] were found to be less likely to be associated with TB than their male counterparts (Table 3). Older adults with no education, compared to those who had primary [AOR = 1.08, 95% CI = 1.06–1.10, *ρ* < 0.05] or secondary/higher education [AOR = 0.65, 95% CI = 0.64–0.67, *ρ* < 0.05], had the lowest odds of being associated with having TB. However, regarding the contextual factors, older women residing in rural locations [AOR = 1.38*, 95% CI = 1.36–1.40] were more likely to be associated with TB than their urban counterparts. Older adults with poor health status [AOR = 12.03*, 95% CI = 11.55–12.52, *ρ* < 0.05] were more likely to be associated with TB than their counterparts with excellent health status (Table 3).

## Discussion

We examined the prevalence and association of HIV and TB status in older adults in South Africa. The study has found a prevalence rate of HIV to be 5.9% and TB to be 2.9% among older adults aged 50 years and above in South Africa. Therefore, this study prevalence was found to be relatively lower than the national prevalence of HIV (13.9%) and TB (estimated incidence of 360,000 cases of active TB in 2019, which is a rate of 615 per 100,000 population) when compared to the general adult population, respectively. The estimated prevalence of tuberculosis (TB) in South Africa in 2018 was 737 per 100,000 population (6, 11). According to the World Health Organization (WHO), South Africa had an estimated incidence of 360,000 cases of active TB in 2019, which is a rate of 615 per 100,000 population (12). However, our findings showed that a higher prevalence of older adults living with HIV was found in Gauteng and KwaZulu-Natal provinces. A higher prevalence of older adults living with TB mainly resided in KwaZulu-Natal and Eastern Cape provinces. Similarly, with cross-province variations, the lowest prevalence of older adults living with HIV and TB was found residing in the Northern Cape Province. Thus, the findings of the cross-province variations, with the lowest prevalence of older adults living with HIV and TB found in the Northern Cape Province were surprising. For instance, the prevalence of HIV among older adults in this study were mostly found in Gauteng and KwaZulu-Natal provinces, however, as reported by other studies, Gauteng was the fifth province with HIV prevalent cases in the general population (28.1%) (2, 3). Also, the findings of this study found out that Northern-Cape town has the lowest HIV prevalence cases among older adults. This finding is not consistent with the previous studies that showed that Western Cape has lower prevalence of HIV in the general population (17.9%) (2, 3).

Another unpredicted example was observed in the prevalence of older adults with TB, and this study findings reported Eastern Cape and KwaZulu-Natal provinces with a higher prevalence of older adults living with TB. This study findings corroborates with the trends in the general population as reported in existing studies (6–8). However, the finding of this study further revealed that Northern Cape province has a lower prevalence of older adults living with TB but this finding does not corroborate with previous trends in the general population as other studies reported Limpopo province with the lowest prevalence cases of TB (7, 8). In many provinces where the older adult population is relatively high, the older adult population should be prioritized under the sustainable development goals and policies adopted from the United Nations global goals (37, 38). The transformational goal of the 2030 Agenda and the commitment to “leave no one behind” demonstrated the importance of including people of all ages in development plans. Since older individuals have an equal right to development, the SDGs must be implemented in accordance with equality, social justice, and human dignity throughout the life span (22). A global population with aging concerns results from a continued decline in fertility rates and increased life expectancy. Importantly, SDG 3 aspires to ensure health and well-being for all by 2030, including a bold commitment to end the epidemics of malaria, AIDS, TB, and other communicable diseases (38). This further leads to achieving health goals and providing adequate access to healthcare for older populations.

Several studies have shown that in South Africa, HIV infections was particularly segregated by population groups: 13.6% of Black Africans have HIV, whereas only 0.3% of Whites have the disease. False traditional beliefs about HIV, has contributed to the spread of the disease, persist in townships owing to the lack of education and awareness programs in these provinces of South Africa. Other social concerns such as sexual violence and local attitudes toward HIV have also amplified the epidemic (33, 34). Also, South Africa bears one of the highest burdens of TB disease in the world. Thus, year on year, the country has a high incidence of TB infections and tens of thousands of South Africans lose their lives owing to TB disease. This is compounded by the prevalence of HIV-TB co-infection and ongoing issues with drug-resistant TB strains which makes treatments more lengthy and more costly. TB disproportionately affects individuals living in abject poverty and poverty in South Africa is becoming more and more problematic as the unemployment rate increases in the midst of long entrenched income inequality. South Africans facing the worst economic hardships are the least likely to access TB diagnosis and treatment, which spotlights the urgent need to focus and improve TB health services in under-served communities and provinces (33, 34). However, TB is an infectious disease caused by *Mycobacterium tuberculosis*, a bacterium that primarily affects the lungs, as it spreads through airborne bacteria released in tiny droplets when an infectious person with active TB coughs or sneezes (33, 34). Particularly, close and prolonged contact with an infectious individuals is necessary for the bacteria transmission, and not all who are exposed to the bacteria develops active TB disease. Several studies have shown that in many cases, the immune system can keep the TB infection dormant, however, those with weakened immune systems, such as people living with HIV, are more likely to be susceptible to developing active TB disease (33, 34).

Similarly, malnutrition is another factor that leads to a weak immune system and a higher risk of developing TB disease. In South Africa, food insecurity is a major cause of malnutrition, alongside with chronic substance abuse which often lead to being underweight as the outcomes. Several studies have shown that nutrition plays a major role in establishing a strong immune system, under-nourishment, including significant loss of fat and muscle mass, is one of the risk factors of TB (34–36). Similarly, another study have indicated that where there is malnutrition, stress, drug addiction, alcoholism, and abject poverty, the presence of TB disease is often seen among vulnerable and high-risk populations (32, 33). Likewise, owing to the poor access to healthcare and no medical aid scheme membership, malnutrition is extremely often before now severe at the stages of diagnosis. The human body requires extra amount of energy, protein, and nutrients to fight off infection, and healthy weight is very important to the efficacy of TB drug treatments. Though, maintaining body mass and promoting weight gain can be challenging as both HIV and TB disease-states interfere with the human body’s ability to absorb food nutrients (5, 34). Our findings were that a comparison of middle-aged older adults in age cohorts of 60–64 years and 55–59 years recorded higher odds of having HIV and TB infections confirming the findings of Bhebhe et al. (39) in Botswana. Several explanations have been given for this association. According to Rodríguez-Sánchez et al. (40), as people age, they become more aware of their purpose in life. As a result, there is a sense of commitment to providing better care for the general well-being of older people. This association was further explained by Rabe et al. (41) in the context of the direct relationship that exists between increasing age and being economically secured. Thus, adults, especially those with HIV and TB, can care for themselves as they age, become financially independent, and can bear the costs of subscribing to medical insurance schemes covering TB-and HIV-related illnesses.

The study found that older adults who had never married or were widowed have higher odds of HIV and TB, confirming similar findings conducted in Kenya (42). Planning for a social and economic shift related to older adults is essential to ensure speedy progress toward achieving the goals outlined in the 2030 Agenda for Sustainable Development. However, trends in population aging are particularly relevant to SDGs in eradicating poverty and ensuring healthy lives and well-being at all ages. Thus, promoting gender equality, productive employment, and decent work for all shows that reducing inequalities between and within countries will support the inclusiveness, safety, resilience, and sustainability of human settlements (38). Similarly, the rapid aging of the population around the globe has presented an unprecedented set of challenges to the aging population, such as shifting disease burden, labor-force shortages, increased expenditure on health and long-term care, lack of savings, and potential issues associated with old-age income security (43). In addition, societal aging can affect patterns of work and retirement, economic growth, the ability of governments, the function of family structures, and ability of communities to provide adequate resources for older adults with HIV and TB, and the prevalence of chronic disease and disability. Studies have indicated the unprecedented rapid growth in the number of older individuals globally, which is a consequential situation where, by the year 2050, almost 22% of the global population will be over the age of 60 years (44, 45). Thus, the changes in demographic trends are becoming a significant concern for legislative bodies, global non-governmental organizations, and communities. This is especially true given that older adults are among the most vulnerable and neglected demographic groups, invisible to those advancing economic and social development (46).

The highest HIV/AIDS prevalence rate found in this study finding was among those who were never married compared to those who are married, while the highest TB prevalence rate was found among older adults who are widowed compared to those who are married. This study findings of HIV/AIDS prevalence rates sought to corroborates with studies that also mentioned higher prevalence rates of HIV/AIDS among never married individuals (47, 48). Also, several studies have confirmed that TB prevalence is increasing among individuals who are widowed (43, 49). Similarly, there are several sub-Saharan African nations where the prevalence of HIV and TB continues to increase, and older adults who had never married or were widowed have a higher likelihood of contracting these diseases (39, 50). This may be because they neglected to purchase health insurance to mitigate their financial, medical, and personal burdens. According to several studies, the prevalence of HIV among newly diagnosed TB patients in South Africa increased progressively over time, rising from 44% in 2006 (51) to 60% in 2010 (52). There is an urgent need for HIV reduction programs in this subgroup of older persons given the high risk of HIV infections among TB patients. According to a study by Zachariah et al. (53), TB patients are more likely to engage in dangerous sexual conduct, such as unprotected sex. All ages, including older persons, are sexually susceptible to HIV transmission in South Africa (54, 55). As one of the HIV prevention methods, it is necessary to persuade older persons with TB to adopt safer sexual behavioral modifications (56). Using condoms correctly and consistently is one of the most effective ways to prevent HIV among sexually active individuals, such as older adults who have never been married or who are widowed, and who may be tempted to experiment with unprotected sexual contact with younger partners there is a dearth of information or data on condom use behavior among older adults with HIV and TB in South Africa. For instance, studies have shown that about 78% of adult TB patients in Ethiopia reported that they had not used condoms, and in Thailand, 42% of HIV-infected TB patients reported never using condoms at all (57). Designing HIV prevention initiatives that promote and encourage condom use among older adults with HIV and TB infections requires an in-depth re-assessment and evaluation of their condom use behavior and the factors that influence their non-use.

According to our findings in this study, there is a significant association between gender and HIV and TB status among older persons. We found a lower chance of having HIV and TB in older female adults when compared to their older male counterparts. This finding agrees with existing studies that reported a significant association between gender and HIV and TB status in older adults (28). In South Africa, given the HIV and TB epidemics among the older population, clear evidence about testing older persons (over 50) becomes necessary. Unfortunately, there are dearth of known studies that has focused on the conditions or situations under which older people test for HIV or their motivations for doing so. Therefore, it is of the utmost importance to understand the decisions and pathways older South Africans living with HIV and TB take to get tested and treated. As such, men may test positive for HIV when their partners receive an HIV or TB diagnosis or treatment (38). As a result, older women—who are more likely to be widowed or divorced—frequently only test positive for HIV when they are symptomatic or are not responding as expected to treatment for opportunistic infections or other non-communicable diseases. According to findings from previous study, South Africans do not request testing in response to risk, instead, older men and women only get tested when a doctor refers them because of symptoms or because of their partner’s health (39, 41). Older women typically show surprise and perplexity upon realizing they are HIV-positive because they do not perceive themselves as at risk of contracting HIV, according to studies done in South Africa (39, 40). The South African government has made many efforts to provide a policy intervention named Universal Test and Treat (UTT), which ensures everyone, including older adults, can go for HIV testing and receive treatment if the test outcome is positive (10). This UTT policy intervention provided many benefits of timely recognition and treatment/management; hence, older adults’ predisposition to test at late stages of HIV and TB illness, as well as their opportunistic infections, decreases the individual and population level benefits of accessing UTT.

Studies have also showed the efficacy of UTT facilities for comprehensive well-being intervention programs anticipated to enhance public responsiveness toward HIV and TB testing (4, 5). However, more studies are required to recognize older adults’ risk and testing behaviors and attitudes so that long-time effective policy programs such as HIV testing messages may reach this neglected and vulnerable population. In our study, older adults with primary and secondary/higher education were less likely to be associated with HIV and TB than non-educated older individuals. This result agrees with the outcomes of a few earlier research (37). The findings of our study may be explained by the fact that educated women are more likely compared to uneducated women to take up insurance for their medical care to avoid costs associated with potential future unforeseen health issues and to develop access to adequate and timely health information regarding HIV and TB infections (22). Even though there is no vaccine or cure for this illness, education has emerged as one of the most economical strategies to stop its spread. In addition, education can save lives by giving individuals the knowledge to reduce their risk of becoming infected (8). Thus, educational programs are developed to improve knowledge, attitude, and practice (KAP) and reduce stigmatization among older adults with HIV and TB (4, 40). It is essential that older people are aware of how to take care of themselves and prevent the spread of TB and HIV diseases. Furthermore, several health demographic studies have proven that education can lead to more accurate health beliefs and knowledge, thus people can develop better lifestyle choices, skills, and greater self-advocacy. As such, education improves skills such as literacy, develops effective habits, and may improve cognitive ability (38). Higher levels of education are also likely to expose older persons to a wide range of information, highlighting the need to maintain a healthy lifestyle. On the other hand, older persons who lack education may not have as much access to health information regarding preventive, curative and management of TB and HIV infections/illnesses (48). This may well justify the decrease possibility of subscribing to medical aid schemes among older adults without formal education.

Our findings that older adults who had no business activities recorded higher likelihoods of HIV/TB infections, which could mean that poverty and unemployment are associated with societal circumstances known to increase both the risk for acquiring and prevalence of HIV and TB, as well as other co-morbidities (48–50). Older adults engaging in business activities gives them economic independence that can assist them take care of the bills that come with medical aid premiums, and in consequence lead to an increased probability of accessing medical attention when needed. The results of some earlier investigations are likewise supported by this finding (49). Thus, several studies have mentioned that HIV and TB are diseases that are embedded in social and economic inequity, as it affects those of lower socio-economic status and disadvantaged communities at a disproportionately high rate. Many studies conducted on HIV and TB and unemployment suggested that for older adults, being without employment may affect their likelihood of contracting HIV and TB as well (13). Thus, employment and wealth index are major critical factors in determining the quality of life for older adults after being affected by HIV and TB (17). We found that older adults residing in rural areas had increased odds of being associated with HIV and TB. This outcome replicates the insistent challenges of social disparities of health across South Africa (22). For instance, health facilities are primarily found in urban areas, and many health practitioners refuse to be posted to rural areas. Access to testing health facilities is therefore usually a big challenge for older adults with HIV and TB illnesses, especially in healthcare facilities where they are afraid of being stigmatized by their health conditions (48). Also, medical aid cover that covers the medical expenses of HIV and TB-related illness constitutes part of the health care facilities, which is mainly aimed at the urban areas. Therefore, it is not unexpected that the odds of coverage will be decreased in the rural areas.

Our results revealed a significant association between health status and older adults with HIV and TB. We found a higher likelihood of poor health status among older adults with HIV and TB than among those with excellent health status. This finding is in accordance with other studies that showed a substantial relationship between older persons with HIV and TB, and their health status (5). Nevertheless, the overall well-being and health of older persons in sub-Saharan Africa, including South Africa, has received little attention from research. Even less is known about the overall well-being of older adult people who have HIV or TB infections or have been adversely affected by the consequences of HIV or TB among their ‘significant others’ (45). In older persons with HIV and TB receiving ART, the persistence of subclinical inflammation has resulted in chronic immune-mediated problems, which are thought to cause cardiovascular dysfunction, end-stage organ failure, and an increase in co-morbid diseases (27). Thus, being older increases health risks, such as having various diseases and being more susceptible to drug toxicity (9). In addition, studies from developed countries have looked into particular issues related to aging in HIV-infected individuals on antiretroviral therapy (ART) (12). Some of these concerns include a deteriorating immunological response to ART, the possibility of clinical complications brought on by ART, the presence of co-morbid illnesses (such as Hepatitis B or C) or risk factors (for example, smoking, alcohol abuse, etc.), and the burden of health conditions brought on with normal aging (50). Furthermore, regardless of whether they are using ART, several studies have observed greater frailty in older HIV-infected adults (49).

However, few contemporary studies have mentioned the health status of HIV and TB-infected older adults in sub-Saharan African countries, including South Africa. South Africa has a paucity of data on older adults with HIV and TB (39, 50, 51). As a result, health issues are increasingly prevalent as people age and, as is to be expected, health deteriorates as people age, first physically and subsequently according to self-reported health and functioning indicators. Also, according to another study, more older women than older males have reported having lower health and functioning body ability (52–55). This was because many women do not have basic knowledge of some health circumstances such as poor vision and hypertension. Our study recorded lower odds of diabetes and cancer in older adults with HIV and TB. Older adults with HIV have an increased risk of health conditions such as diabetes, dementia, osteoporosis, frailty, and some cancers. Other studies, however, recorded that individuals with HIV infections are more likely to have Type 2 diabetes, and some HIV medicines may increase the risk of Type 2 diabetes in persons with HIV infections (10, 43). In addition, older adults infected with HIV are at an elevated risk of developing cancer. Nevertheless, some recent research has demonstrated that women with HIV have a markedly elevated risk of cervical cancer (38, 45). Therefore, it is imperative that HPV vaccination and cervical cancer screening should be undertaken by women living with HIV (18–20).

Regarding medical aid schemes, our study revealed a significant association between medical aid schemes and older adults having HIV and TB. The likelihood of an older person having HIV and TB and no medical insurance was higher, according to our research. Our study findings revealed that older adults with HIV and TB without medical aid coverage had no business activities, were from the poorest households, and residing in rural provinces. Studies have shown that wealthier older adults are more likely to afford and opt for medical aid premiums and access private health facilities where they can have quality healthcare provided to them for their HIV and TB status (11, 17). In most sub-Saharan African countries, such as in South Africa, medical aid coverage usually applies to private health facilities, while public health facilities usually treat those without medical aid (37, 43). Thus, a medical aid scheme gives people with HIV and TB access to appropriate HIV medical care, particularly HIV treatment called antiretroviral therapy (ART), which helps older adults with HIV and TB to stay healthy and prevent transmitting HIV and TB to others (8, 23). However, other studies reported decreased odds of older persons without HIV and TB infections enrolling in medical aid schemes (28, 42). This may explain why we found that older adults with HIV and TB illnesses and no business activities were more likely to be covered by the medical aid scheme compared to those with business activities and having reported HIV and TB in our multi-level regression Model IV, which was a multi-level regression analysis. Thus, several authors have shown that older adults with HIV and TB illnesses who own medical aids can illustrate the positive impact of health advocacy efforts for an aging population to raise the general population’s knowledge of the importance of consistently having easy utilization of health care facilities and medical care (56, 57).

Government and civil organizations should make sure that older people are not only seen as important resources who significantly contribute to the economy, but also as dependents who require substantial care and assistance (58). Political will and commitment are vital in ensuring that the basic principle of the 2030 Agenda of leaving no one behind fully includes older adults (28, 59). These commitments include waivers from paying annual payments and free health plans incorporated into health insurance programs. Such interventions can make the lives of older persons more valuable in society in general, as well as in their grassroots communities.

### What is the way forward? The need to escalate the scientific and political attention to aging and health

The rapid growth of aging population and the relatively slow control of the concomitant HIV and TB epidemics among older adults living with HIV are two of the most significant challenges facing South Africa in the post-apartheid era. Similarly, HIV is still persistently spreading, and TB has been deemed a national emergency, which is made worse by increased drug resistance and co-infection with HIV. However, prior to recent times, the South African government’s approach to these health conditions, including among the older population, has been marked by denial, lack of political will, and poor implementation of policies and programs. To properly tackle the HIV and TB burden among older adults in South Africa, there is a dire necessity to escalate scientific and political attention to the health problems associated with aging and health conditions, especially among older adults living with HIV and TB diseases. We look at these two specific levels below:

#### Scientific attention

- Why is studying aging and older adults from Sociological and Demographical perspectives important? Of course, biological and psychological factors affect this process of aging over the life course. However, a sociological and demographical perspective significantly contributes to our understanding of aging by clarifying how social, economic, and political forces shape the aging experience in our contemporary societies.
- Understanding the information and tackling misinformation and disinformation about the aging population and health is part of policy development and implementation. Continued research, key policy modifications and research investment collaborations could significantly increase health outcomes for older persons with HIV and TB by increasing support and treatment accessibility for older adults.
- Gerontological research is key in understanding how different older age cohorts experience HIV and TB and other comorbidities, yet there is a missing link. In South Africa, South African medical professionals are insufficiently to deliver medical services to older adults and poorly equipped, as most specialist in gerontology are mostly found working in academic communities. Also, medical training institutes have yet to introduce sub-specialist with sub-specialization in gerontology that conjointly offer aged care and HIV and TB, and dearth of skilled consultants on communicable illnesses in the field of gerontological medical specialty, presenting difficulty to the medical system in the future. So primary care practitioners will probably be treating older people with HIV and TB, as they currently do. Thus, treating and caring for older adults with HIV and TB is difficult and multifaceted. Therefore, gerontological research institutes/ departments should be built, and gerontological courses should be included in the university curriculum for medical practitioners to find the missing link and space for its contents.

#### Political attention

- South Africa has adopted a comprehensive policy and overall legislation for aged people, adapted for domestic use from the United Nations Principles for aging population. The South African legislature looks forward to fostering aging personalities and attending to their issues and challenges. However, this government initiative must back a multifaceted plan that implements the social determinants of HIV and TB among older adults in South Africa, as well as socioeconomic concerns that affect this high-risk population.
- In African nations, including South Africa, the health care infrastructures are ill-equipped to handle the distinctive medical therapies and health needs of those who are older adult living with HIV and TB presently. The long-term health effects of ART use and several concurrent medical conditions in older adults with HIV and TB are still being discovered. There should be political will and commitment from the government to increase sensitization to support the awareness of the aging population and their health.
- Medical training of health care providers in meeting the needs of older adults is very important, and social marketing campaigns and relevant government stakeholders should confront the stigmatization of older adults with HIV and TB.
- Importantly, as society ages, greater demands for housing and infrastructures to meet the evolving demands of older persons should be included in the policy driven initiatives for the older persons. In order to improve the housing and amenities available for people in older years, governments should design a strategic approach in meeting the housing needs of an aging population.

### Strengths and limitations of the study

This study’s main strength is its use of nationally representative data to evaluate contextual and individual-level characteristics related to older persons living with HIV and TB in South Africa. Advanced statistical models that consider clusters in the sample were also used in the study. We also used the present version of the GHS of South Africa. This ensures that our findings reflect the recent representativeness of the situations of the older population with HIV and TB. Furthermore, the survey data collection utilized in this study have highlighted high-standard methodological techniques, as well as highly trained research assistants, thereby ensuring the high-quality data collected and utilized. Besides, we used higher-order statistics such as multi-level binary logistic regression, in order to conduct our research. This ensured a thorough review of the data and took validity concerns into account.

Considering that it has some limitations, the study design was cross-sectional and, therefore, causal interpretation cannot be deduced. For instance, it is significant to consider that the cross-sectional design selected for collecting the GHS data restricts the chance to draw any causal inferences about the associations between the variables under investigation. Also, methodological weaknesses can partly explain some of the variations found in the findings of the prevalence of HIV and TB among older adults across the provinces in South Africa. Further, given that older persons with HIV and TB reports retrospectively, the study may be impeded by recall bias, a problem that frequently plagues the GHS. Thus, data are often probe by asking further questions in order to reduce potential bias, as follow-up questions are often asked for instance, ‘have you had/has TB’ and the participant said yes, then a follow up question on ‘are you using medications’ and if the person said yes, ‘which of the medications are you using’, and the participants will tell them. Therefore, a probing question is often used in regard to potential bias during data collection. Most importantly, the 2019 GHS surveys covered respondents who reported HIV/AIDS and TB separately, but HIV/TB co-infection were not included in the 2019 GHS datasets as at the time of carrying out this study research. However, there are clinical datasets on HIV/TB co-infection outside the 2019 GHS surveys, which are not made available owing to ethical practices and national policy/laws (South Africa’s HIV and AIDS policy and legislation). Thus, the statistical information on the prevalence on HIV/AIDS and TB co-infection in older adults was not included in this study, hence it is outside the scope of this study.

## Conclusion

Despite the fact that South Africa is currently going through a rapid demographic change, the nation is ill-equipped to deal with the issues pertaining to wellbeing circumstances and healthcare systems associated with older adults having been diagnosed with HIV and TB. It is imperative for all stakeholders to be proactive in responding to the demographic shift and seize the possibility to study aging and its difficulties from the perspective of worldwide aging population. As South Africa moves toward achieving the SDG 3 targets of ensuring healthy lives and promoting well-being for all ages, including older adults, it is vital to incorporate interventions that will address the needs of an aging population. Therefore, given the exclusive settings, the South African government should go into unique resolutions based on the country’s conditions and encourage everyone in society to contribute to active, healthy, and productive aging. The integration of medical and care systems needs to be enhanced. Promoting the geriatric care system could be established by encouraging all cooperation between medical and geriatric care institutions. To enable older adults to maximize their potential and values, the government and civil societies must create age-friendly infrastructures and repair existing homes and amenities to make them suitable for older persons to occupy. Additionally, reinforcing the top-level planning and creating a pattern of government-led multi-sectoral cooperation with the social contribution of older adults in providing welfare schemes to carter for this high-risk population.

## Data availability statement

The datasets presented in this study can be found in online repositories. The names of the repository/repositories and accession number(s) can be found in the article/supplementary material.

## Ethics statement

The protocol was approved by the Ethics Committee of the University of Cape Town and Statistics South Africa of the GHS Programme/project (Reference ID: zaf-statssa-ghs-2019-v1). Procedures and questionnaires for the standard General Household Survey (GHS) have been reviewed and approved by the 2019 General Household Survey (ghs-2019-person-1.0-stata11) Institutional Review Board (IRB). All human subjects gave their written informed consent for inclusion before participating in the study. Also, the study was conducted in accordance with the Declaration of Helsinki, as well as with the relevant ethical guidelines and regulations. We obtained approval to use the 2019 GHS datasets from the GHS website (https://doi.org/10.25828/vtvj-pv21).

## Author contributions

MEA: Conceptualization, Project administration, Data curation, Formal analysis, Software and Writing – original draft. MEA and GNO: Investigation and Methodology. MEA and GNO: Resources and Validation. MEA and GNO: Supervision and Visualization. MEA and GNO: Writing–review & editing. ESI: Postdoc advisor. All authors contributed to the article and approved the submitted version.
